# Supplementary material for: Repositioning Trimebutine Maleate as a Cancer Treatment Targeting Ovarian Cancer Stem Cells
Source: Cells. 2021 Apr 16;10(4):918. doi: 10.3390/cells10040918 (PMC8072797; doi:10.3390/cells10040918)
Supplement: Supplementary file 1 [file cells-10-00918-s001.zip › cells-1137935-supplementary.pdf]

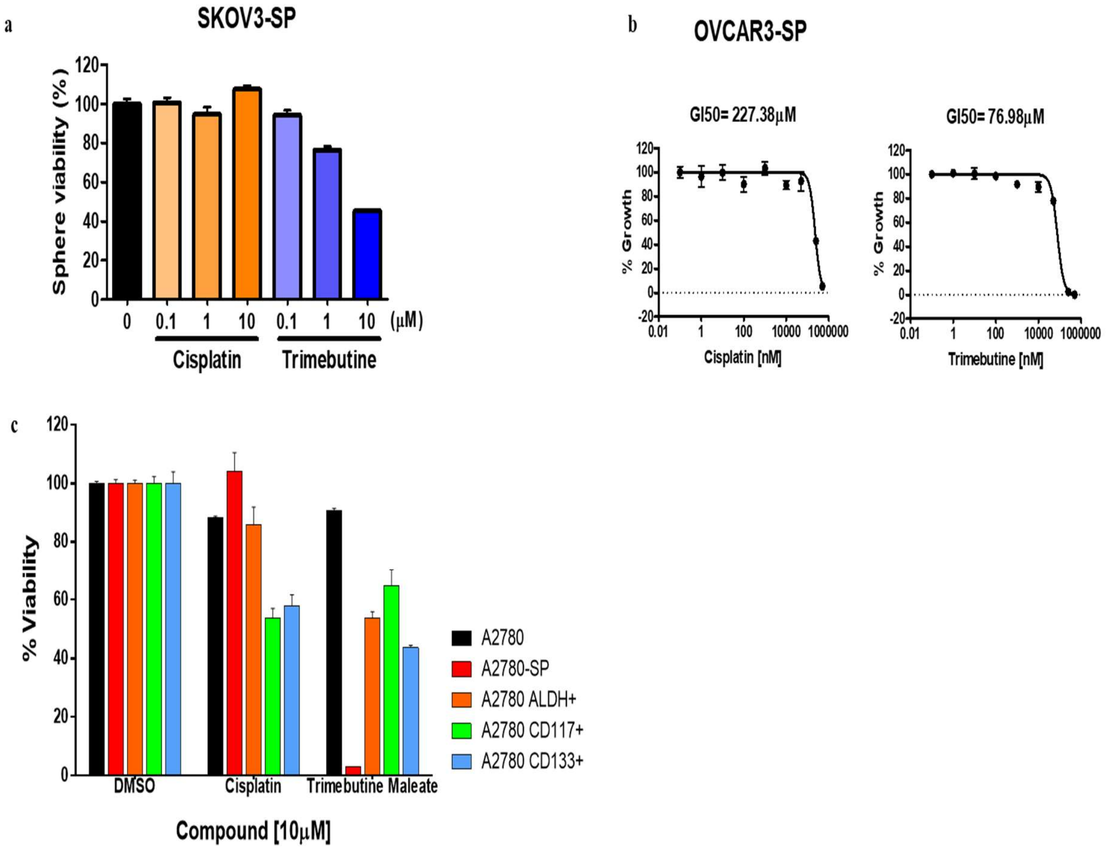

Figure S1. Cell viability effect of TM on various ovarian CSCs. (**a**) ATP-based cell viability assay on SKOV3-SP cells derived by s from SKOV-3 cells of TM or cisplatin with various doses. (**b**) ATP-based cell viability assay on OVCAR3-SP cells derived from OVCAR3 cells of TM or cisplatin with various doses. (**c**) ATP-based cell viability assay on sorted cells by ovarian CSC marker such as ALDH, CD117 and CD133 of TM or cisplatin at 10 uM.

a

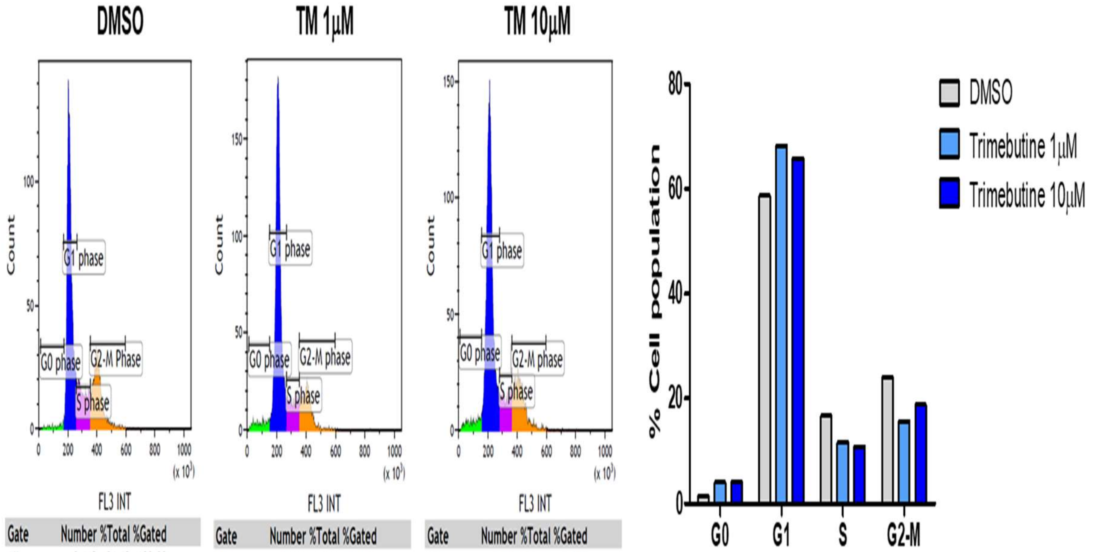

DMSO

Gate	Number	%Total	%Gated
All	8,112	81.12	100.00
G0 phase	113	1.13	1.39
G1 phase	4,762	47.62	58.70
G2-M Phase	1,946	19.46	23.99
S phase	1,357	13.57	16.73

TM 1μM

Gate	Number	%Total	%Gated
All	9,998	99.98	100.00
G0 phase	408	4.08	4.08
G1 phase	6,823	68.23	68.24
G2-M phase	1,572	15.72	15.72
S phase	1,169	11.69	11.69

TM 10μM

Gate	Number	%Total	%Gated
All	9,999	99.99	100.00
G0 phase	405	4.05	4.05
G1 phase	6,574	65.74	65.75
G2-M phase	1,880	18.80	18.80
S phase	1,092	10.92	10.92

Figure S2. Cell cycle arrest analysis by TM on A2780-SP cells. (**a**) Cell cycle was analyzed by propidium iodide (PI) staining in A2780-SP cells 24 h after TM treatment (1, 10 M). The percentage of cells in the G0, G1, S, and G2-M phase in independent duplicate cultures were plotted (right panel). Bars, ±SD of duplicate cultures.

a

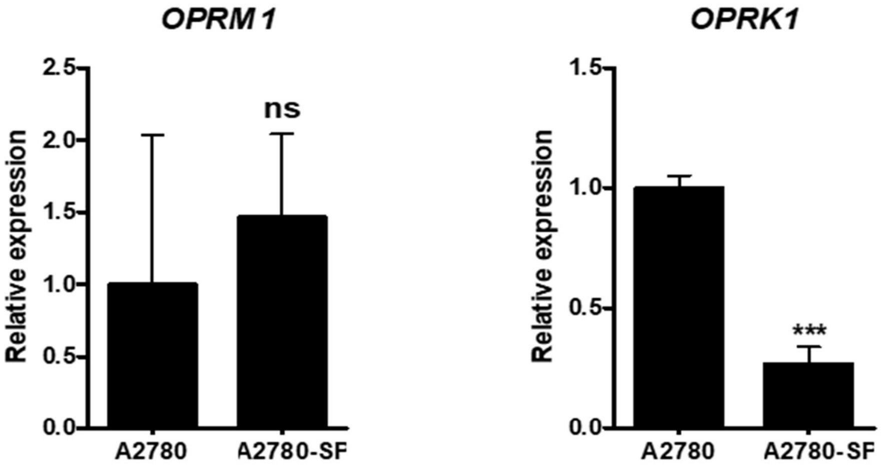

b

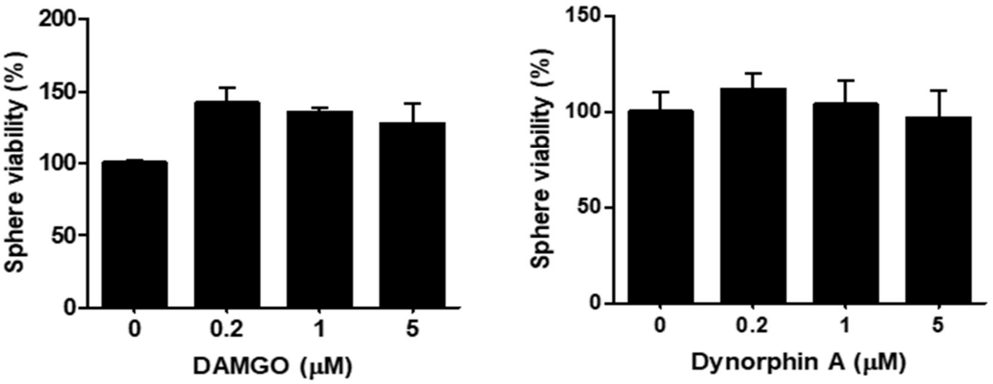

Figure S3. Opioid receptors on ovarian CSCs. (**a**)Relative mRNA expression of u-opioid receptor and k-opioid receptor on A2780-SP cells compared with A2780 cells. (**b**) Cell viability effect of opioid receptor agonists such as DAMGO and dynorphin with indicated concentration on A2780-SP cells.

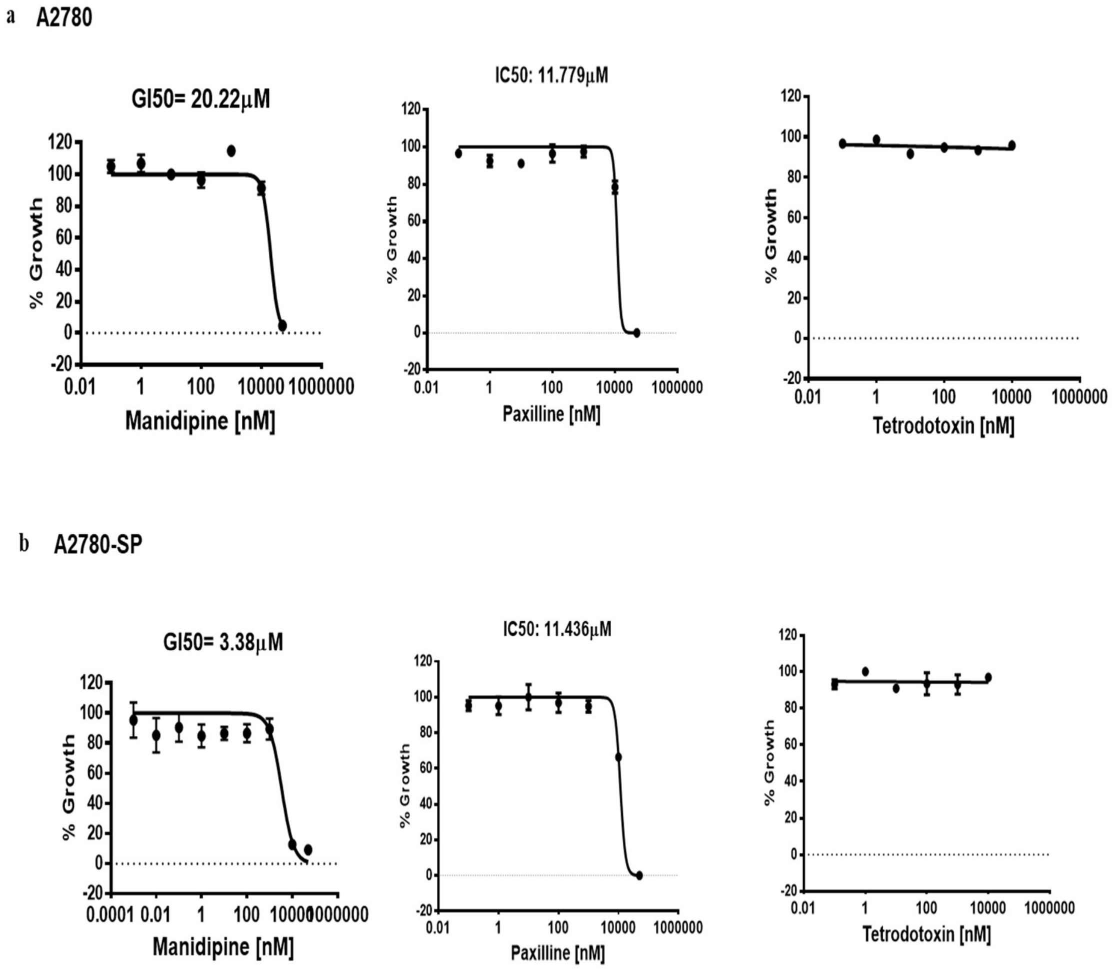

Figure S4. Cell growth inhibition effect by inhibition of calcium channel, BKCa channel or Na channel solely in ovarian cancer cells or ovarian CSCs. (**a**) ATP-based cell viability assay on A2780 cells of mannidipine as a calcium channel blocker, paxilline as a BKCa channel blocker and tetrodotoxin as a sodium channel blocker with various doses. (**b**) ATP-based cell viability assay on A2780-SP cells of manidipine, paxilline and tetrodotoxin with various doses.

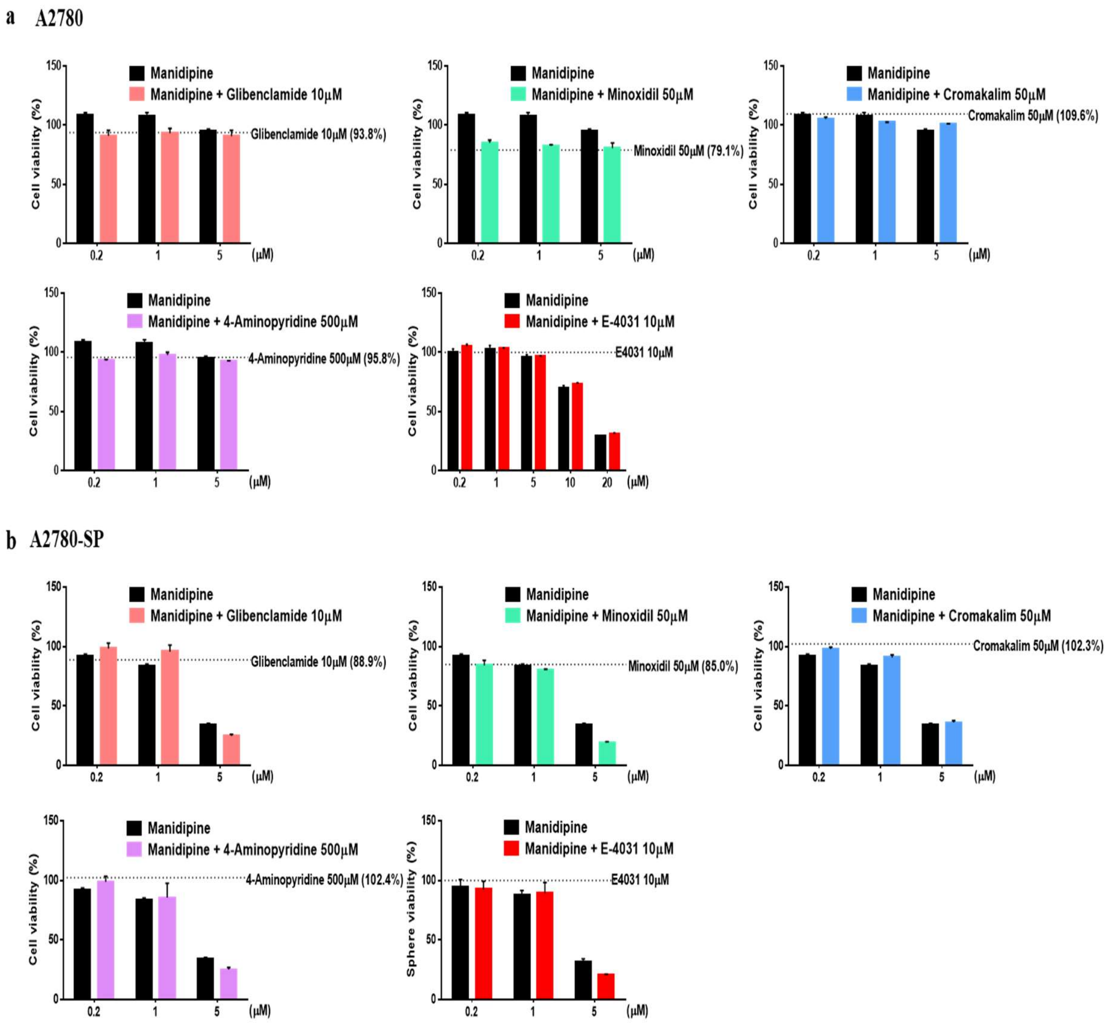

Figure S5. The simultaneous inhibition effect of other potassium channels besides BKCa with calcium channel on ovarian cancer cells or ovarian CSCs. (**a**) ATP-based cell viability on A2780 cells by co-treating manidipine at indicated concentration with various potassium channel blocker compared to treating manidipine at indicated concentration alone. 10 uM of glibenclamide as Kir channel blocker, 50 uM of minoxidil, 50 uM of cromakalim as Kir channel opener, 500 uM of 4-aminopyrimidine as Kv channel blocker and 10 uM of E-4031 as hERG channel blocker were used. (**b**) ATP-based cell viability on A2780-SP cells by co-treating manidipine at indicated concentration with various potassium channel blocker as mentioned earlier compared to treating manidipine at indicated concentration alone.

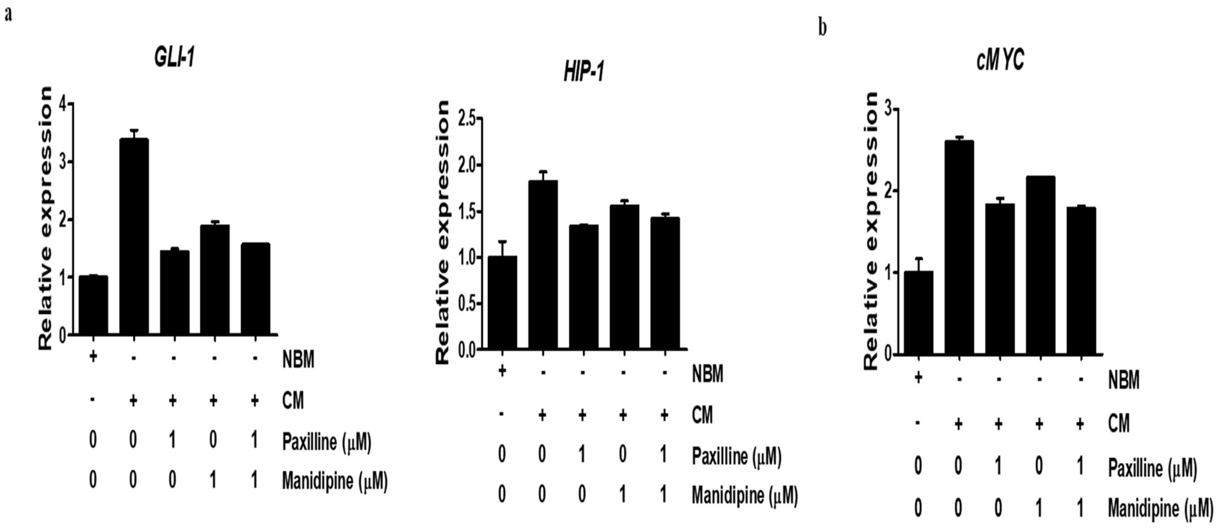

Figure S6. The change of genes related to Hedgehog and Notch pathway by simultaneous blockade of Ca channel and BKCa channel on CSC culture of ovarian CSCs.Relative mRNA expression change of (**a**) Hedgehog signaling such as GLI-1 and HIP-1 and (**b**) Notch signaling related genes such as c-MYC by co-treating 1 uM of manidipine with 1 uM of paxillin compared to treating each independently on CSC culture of A2780-SP cells.
